# COVID-19 Vaccine Acceptance and Access Among Black and Latinx Communities

**DOI:** 10.1001/jamanetworkopen.2021.28575

**Published:** 2021-10-13

**Authors:** Lilanthi Balasuriya, Alycia Santilli, Jennifer Morone, Jessica Ainooson, Brita Roy, Anuli Njoku, Andrea Mendiola-Iparraguirre, Kathleen O’Connor Duffany, Bernard Macklin, Jackson Higginbottom, Celina Fernández-Ayala, Genesis Vicente, Arjun Venkatesh

**Affiliations:** 1 Yale National Clinician Scholars Program, Yale University School of Medicine, New Haven, Connecticut; 2 Community Alliance for Research and Engagement, New Haven, Connecticut; 3 Veterans Administration Health Services Research and Development Center for the Study of Healthcare Innovation, Implementation, and Policy, West Haven, Connecticut; 4 Yale School of Public Health, New Haven, Connecticut; 5 Department of Internal Medicine, Yale University School of Medicine, New Haven, Connecticut; 6 Department of Public Health, College of Health and Human ServiceSouthern Connecticut State University, New Haven; 7 Department of Psychiatry, Yale University School of Medicine, New Haven, Connecticut; 8 Department of Emergency Medicine, Yale University School of Medicine, New Haven, Connecticut; 9 Center for Outcomes Research and Evaluation, Yale University School of Medicine, New Haven Connecticut

## Abstract

**Importance:**

Black and Latinx communities have faced disproportionate harm from the COVID-19 pandemic. Increasing COVID-19 vaccine acceptance and access has the potential to mitigate mortality and morbidity from COVID-19 for all communities, including those most impacted by the pandemic.

**Objective:**

To investigate and understand factors associated with facilitating and obstructing COVID-19 vaccine access and acceptance among Black and Latinx communities.

**Design, Setting, and Participants:**

This community-partnered qualitative study conducted semistructured, in-depth focus groups with Black and Latinx participants from March 17 to March 29, 2021, using a secure video conferencing platform. Participants were recruited through emails from local community-based organizations, federally qualified health centers, social service agencies, the New Haven, Connecticut, Health Department, and in-person distribution of study information from community health workers. A total of 8 focus groups were conducted, including 4 in Spanish and 4 in English, with 72 participants from a diverse range of community roles, including teachers, custodial service workers, and health care employees, in New Haven, Connecticut. Data were analyzed from March 17 to July 30, 2021.

**Main Outcomes and Measures:**

Interviews were audio-recorded, transcribed, translated, and analyzed using an inductive content analysis approach. Themes and subthemes were identified on the acceptability and accessibility of the COVID-19 vaccine among participants who identified as Black and/or Latinx.

**Results:**

Among 72 participants, 36 (50%) identified as Black, 28 (39%) as Latinx, and 8 (11%) as Black and Latinx and 56 (78%) identified as women and 16 (22%) identified as men. Participants described 3 major themes that may represent facilitators and barriers to COVID-19 vaccinations: pervasive mistreatment of Black and Latinx communities and associated distrust; informing trust via trusted messengers and messages, choice, social support, and diversity; and addressing structural barriers to vaccination access.

**Conclusions and Relevance:**

The findings of this qualitative study may impact what health care systems, public health officials, policy makers, health care practitioners, and community leaders can do to facilitate equitable uptake of the COVID-19 vaccine. Community-informed insights are imperative to facilitating COVID-19 vaccine access and acceptance among communities hardest hit by the pandemic. Preventing the further widening of inequities and addressing structural barriers to vaccination access are vital to protecting all communities, especially Black and Latinx individuals who have experienced disproportionate death and loss from COVID-19.

## Key Points

**Question:**

What factors are associated with facilitating and obstructing COVID-19 vaccine acceptance and access among Black and Latinx communities?

**Findings:**

This qualitative study of 72 participants who identified as Black and/or Latinx identified 3 themes to understanding factors associated with facilitating or obstructing COVID-19 vaccination: pervasive mistreatment of Black and Latinx communities and associated distrust; informing trust via trusted messengers and messages, choice, social support, and diversity; and addressing structural barriers to vaccination access.

**Meaning:**

These findings suggest that community-informed insights may inform health care strategies to maximize vaccine acceptance and access in communities hardest hit by the COVID-19 pandemic.

## Teaser

This qualitative study examines factors associated with facilitating and obstructing COVID-19 vaccine access and acceptance among Black and Latinx communities.

## Introduction

More than 99% of deaths from COVID-19 are now among unvaccinated individuals.^1,2^ Black and Latinx communities experience 2-fold the rate of death due to COVID-19 compared with their White counterparts, and factors rooted in structural racism continue to impede equal access to health for all.^3,4^ Striking racial and ethnic disparities also exist in COVID-19 vaccination rates and vaccine access.^5,6,7^ As of July 2021 in the US, among individuals who have received 1 dose of the vaccine, 58.9% were White, 16.1% Hispanic, and 9.3% were Black, which lags behind national racial and ethnic representation.^8^

Data continue to show lower vaccination coverage in Black and Latinx communities, even as the supply of COVID-19 vaccines outpaces demand.^8,9,10,11,12^ Additionally, COVID-19 cases continue to increase as variants surge and vaccination rates stagnate, making it imperative to understand barriers to vaccine acceptance and access.^8^

While summary statistics and aggregated process data are ubiquitously available, these data merely reflect challenges without providing deeper community-informed insights into the drivers associated with vaccination disparities and the approaches necessary to overcome such obstacles. Additionally, studies have shown that attitudes and perceptions regarding COVID-19 vaccines are variable. Thus, it is imperative to understand community perspectives reflective of the current state of affairs in which 3 COVID-19 vaccines exist. Accordingly, our study aims to investigate and understand what factors are associated with facilitating and obstructing COVID-19 vaccine acceptance and access among Black and Latinx communities to directly inform strategies to improve vaccination rates.

## Methods

The Yale Institutional Review Board deemed this study exempt from approval because participation would not put the participant at risk if responses were shared outside the research, per institutional policy. Informed consent was obtained prior to each focus group. This study is reported following the Consolidated Criteria for Reporting Qualitative Research (COREQ) reporting guideline.

### Study Design

We conducted a community-partnered qualitative study with participants from Black and Latinx communities in New Haven, Connecticut, as part of a Centers for Disease Control and Prevention Racial and Ethnic Approaches to Community Health grant. This grant was provided to the Community Alliance for Research and Engagement (CARE) to address disparities in COVID-19 vaccinations. We partnered with 3 key community organizations and stakeholders, including CARE, the New Haven Health Department, and Cornell Scott Hill Health Center (a federally qualified health center). Through weekly online meetings from February to April 2021, representatives from each group aided in adapting the interview guide, creating a codebook, participant recruitment, and interpretation of findings. The interview guide (Box), coding, and thematic analysis were also informed by the World Health Organization (WHO) Measuring Behavioral and Social Drivers of Vaccination Increasing Vaccination model.^13^ The interview guide was pilot tested and adapted by our community partners to ensure questions were informed by the community and asked in plain language.

Box. **Interview Guide**

1. What are your thoughts and feelings about the COVID-19 vaccine?
2. If you got a call today that someone was offering the vaccine, what would you think about getting it or not getting it?
3. What, if anything, has been helpful in learning more about the vaccine?
4. Where do you get trusted information from in your community?
5. What, if any, challenges do you, your family, or people you know face in getting the COVID-19 vaccine?
6. Is there anything else you would like to add that we might not have asked you about?

### Data Collection

We held 8 semistructured in-depth focus groups (4 in Spanish and 4 in English) over a secure video conferencing platform (Zoom; Zoom Video Communications) between March 17 and March 29, 2021. At that time in New Haven, individuals older than 44 years were eligible for vaccination. To remove technological barriers, participants were able to join by video or phone. Focus groups consisted of 3 to 12 participants and lasted 60 to 90 minutes, and participants were compensated with $30 gift cards. Informed consent was obtained before each focus group, and participants were aware of the study goals.

Focus groups were used because they are a valuable tool for gathering information on attitudes and beliefs about behaviors.^14^ Interactions between participants were also a valuable way to generate data on understanding behaviors, particularly where sociocultural factors may influence health behaviors.^14^ Given the timeliness of the COVID-19 pandemic and our project being embedded within a community vaccination effort, focus groups afforded us the ability to collect a higher quantity and breadth of response on an accelerated timeline necessary to inform community vaccination efforts.

Groups were conducted in English (by L.B., J.A., A.N., and B.M.) and Spanish (by L.B., A.M., C.F.-A., and G.V.), audiotaped, and professionally transcribed in their respective languages. Owing to technical challenges, 1 group was not audiotaped; this interview was analyzed from contemporaneous notes, but it was not used for direct quotes. Spanish focus groups were professionally translated to English. Meetings occurred with the study team and community partners 1 to 2 times a week to review the data and ensure that thematic saturation was reached prior to concluding focus groups. Thematic saturation was defined as when a thorough understanding of participants’ perspectives were represented, and no new themes were subsequently found.^15^

### Participants and Setting

Participants were recruited through emails from local community-based organizations, federally qualified health centers, social service agencies, the New Haven Health Department, and through in-person distribution of study information by CARE community health workers. Eligible participants were current New Haven residents identifying as Black and/or Latinx. Those who did not meet this criteria were unable to participate. A convenience sampling was used.

### Data Analysis

First, a preliminary codebook was made a priori based on the WHO Measuring Behavioral and Social Drivers of Vaccination Increasing Vaccination model by a psychiatrist (L.B.) a nurse scientist (J.M.), a vaccination outreach research assistant (J.A.), a social worker and researcher (A.S.), and a community health worker (C.F.-A.). This codebook was adapted and informed by our community partners as coding progressed. Coding and analyses were performed using inductive content analysis, in which essential concepts from interview data were iteratively coded and compared to extract themes.^16^ Three of us (L.B., J.M., and J.A.) independently performed line-by-line inductive coding to identify initial codes using the online software Dedoose, then collectively analyzed codes, grouping similar codes into subthemes and then larger themes.^16^ The final list of subthemes and themes were agreed on by majority consensus among authors (L.B., A.S., J.M., and J.A.). To ensure the themes and subthemes accurately represented the data and the community’s experience, triangulation was used, in which the we discussed and examined final themes with community partners.^17^

## Results

A total of 79 participants agreed to take part in the study, of whom 7 did not attend and were categorized as nonparticipants. Our final study sample was 72 participants, with 36 (50%) identifying as Black, 28 (39%) as Latinx, and 8 (11%) as Black and Latinx; 56 participants (78%) identified as women and 16 (22%) identified as men (Table 1). Participants were largely from 3 age categories: 50 to 64 years (34 participants [47%]), 30 to 49 years (24 participants [33%]), and 18 to 29 years (13 participants [18%]). Employment backgrounds reflected the diversity of the New Haven community, including teachers, custodial service workers, and health care workers. Vaccination status of participants is noted in Table 1.

**Table 1.** Demographic Characteristics and Vaccination Status of Study Participants

Participant characteristics	Total (%) (n = 72)
Language of focus group attended	
English	46 (64)
Spanish	26 (36)
Sex	
Women	56 (78)
Men	16 (22)
Age category, y	
18-29	13 (18)
30-49	24 (33)
50-64	34 (47)
≥65	0 (0)
Undisclosed	1 (1)
Race and ethnicity	
Hispanic	28 (39)
Hispanic Black	8 (11)
Non-Hispanic Black	36 (50)
Vaccination status	
Fully vaccinated	16 (22)
Started vaccination	8 (11)
Planning to get vaccinated	32 (44)
Not planning to get vaccinated	9 (13)
Unsure	6 (8)
Undisclosed	1 (1)
Employment	
Administrative support	6 (8)
Homemaker	2 (3)
Health care worker	5 (7)
Counselor	3 (4)
Social worker	2 (3)
Maintenance and custodial service worker	6 (8)
Research	3 (4)
Community worker or case manager	8 (11)
Student	4 (6)
Teacher or childcare worker	8 (11)
Unemployed	6 (8)
Retired	2 (3)
Disability	1 (1)
Other^a^	11 (15)
Undisclosed	5 (7)

^a^ Other category includes food services, transportation services, factory workers, residential assistants, insurance verifiers, and customer services workers, among other employment.

Identified themes centered around trust and addressing barriers to vaccine access. They were categorized into 3 key themes: (1) pervasive mistreatment of Black and Latinx communities and associated distrust; (2) informing trust via trusted messengers and messages, choice, social support, and diversity; and (3) addressing structural barriers to vaccination access. Table 2 lists illustrative quotes representative of these major themes and subthemes.

**Table 2.** Study Themes, Subthemes, and Illustrative Quotes From Participants on COVID-19 Vaccine Acceptance and Access

Themes and Subthemes	Illustrative Quotations	Focus group
**Pervasive mistreatment of Black and Latinx communities and associated distrust**		
The lasting legacy from historical mistreatment	“The Tuskegee experiment information in your head…that actually pulled a certain group of Black Americans and did this to them…are they setting certain vials away…to give when a Black person sits down?”	1
	“Specifically, experimentation on Black people, which we know has happened.”	2
Disparate death	“People die from childbirth, regular colds…they’re just ignored…so, I feel that’s a huge part of moving with getting this vaccine too.”	3
	“Black people being overly affected and dying because of the poverty that we live in and all the other social illness from the lack of health care. So if that’s something that we can, basically, make the decision to answer their questions and let them make the best decision for themselves.”	1
	“Knowing a lot of disparities that are with African Americans in health and seeing just the different reactions that people have with COVID[-19]—someone could be 100% healthy and pass away, while someone has preexisting condition, and nothing happen—I feel like there’s just as much risk in allowing yourself to kind of get COVID[-19] as much as getting a vaccine that came out kind of quick.”	3
Experiences of your voice being “thrown away” and ignored	“I had to fight my way to get certain things done for my health.…They don’t take you seriously sometimes…that kind of contributes to the fear of wanting to get vaccines, wanting to do new medical things because it’s like you’ve been put in so many different ways before in the past that you really just don’t trust it.”	4
	“Black women, when it comes to going to a hospital, it’s a fear.…They’ll tell somebody they know 100% what they’re going through and will get it undermined or get their opinion thrown away.…Black voices are regularly not listened to.”	3
**Informing trust via trusted messengers and messages, choice, social support, and diversity**		
Information from trusted messengers	“People who know the community, people who know me, I think that’s the deciding factor that would make me actually want to get it, but definitely not just any doctor.”	4
	“What gave me a lot of confidence in making the decision of whether to want to get it, is that my niece, I tell you, is a specialist and she told me that there are many studies that they did long before looking for the option of give a vaccine.”	5
	“Well, for me, I believe that religious leaders...I think they would listen more to that person.”	7
Consistent messaging	“It’s all confusing because none of them have the same message…makes it harder to believe anything.”	2
	“Conflicting messages can be very confusing…it also adds to the mistrust.”	2
	“It is not so much just television anymore, because right now on the internet you can see more information. Because I’ve seen that Telemundo says one thing, but in reality something else is happening.”	5
Fact-based information	“I think the data that they’ve been giving on the news has been very helpful about the number of vaccines [that] have been given and the reduction in hospital stays…they’re giving us good data showing us that the vaccine is helping.”	2
	“We need to develop a platform where the positive part of the vaccine can be spreading because there’s so much negative in social media.”	3
Transparent and continued communication with the community	“I think that direct conversation about the myths is good.”	2
	“I feel like there’s just that level of transparency that’s needed between healthcare professionals and providers to communities like this.”	3
	“The more we can have this discussion and just be honest with one another, I think the better it’s going to be.”	3
Increasing confidence through choice	“There was a list of the different vaccines, and I checked the one that I wanted.…I had a choice definitely for what I signed up for.”	2
	“They didn’t just say okay you go to this person. When the lanes opened up…they were like, just pick a lane…it just made me feel relieved that I had a choice of which lane I could go to. It wasn’t just like go to the back of the room.”	1
“Go With You”: The power of social support	“They scared to go there by themselves but if they see a neighbor go with them…I see somebody else is in the same boat as I am, then I don’t feel so scared.”	1
	“I want to go with you…where it would give her some strength and some confidence to walk up in there and know that they’re going to be honest, you know what I’m saying?...But just to let her know that there’s a sister or somebody right there next to her.”	4
	“I was very suspicious, but my 18-year-old son already took it.…And he said to me: ‘You know what mommy? You don’t have to be afraid, because this is like when there were other types of vaccines. And only vaccines are going to save us.’”	5
	“My brothers and my sisters got vaccinated, well, they spoke to me. That helped me make the decision to get vaccinated too.”	7
Reassurance in seeing diversity	“Was it just going to be all Black folks in there? And was I going to get back to that thought of, okay they’re trying to do something to us, or that we got the contaminated vials....But when I got in there, seeing the diversity it made me feel better.”	1
	“I think for me, it would be because it would be other Black people actually getting vaccinated....There is power when you see actually someone who looks like you.”	3
	“It was kind of difficult for me to go inside because of the rumors and bad things that I heard about the vaccine. Then when I went inside and seen all the policemen, doctors all in line.…And I’m looking like, okay…must be for real.”	
**Addressing structural barriers to vaccination access**		
“Cutting the Line” and vaccine supply access	“You hear about the White people that are going to the Black and Brown communities and cutting the line. They never be in that community otherwise but to get vaccinated.”	2
	“And one thing people have to look at now, who’s some of the people in front of the line that want the vaccine?…I heard that people coming from Greenwich to New Haven to get the vaccine.”	1
“Going in Circles”: sign-up fatigue	“The numbers that they were giving were not working numbers…they kept going around in circles.”	2
	“Finally, when you get it, you go through the process, you follow all these steps, and when you get to the end, it says, ‘pick a time,’ and it goes, ‘no time available.’”	4
	“And I asked her if she had been able to get vaccinated elsewhere, and she said she was trying to call and they either didn’t call her or they didn’t have the slots available while she’s not working.”	8
No insurance? No worry!	“If you do have insurance, great, but if you don’t, it’s not a worry in back of peoples’ heads.”	3
	“People are afraid to go get vaccinated, because many think that they are going to be charged to their health insurance.”	8
“It’s Right Here”: Schools, workplaces, and community partnerships as facilitators	“It was super easy for me because the clinic was at my school…it’s right here, no excuse.”	4
	“My work helped me, because they sent the initial email so that we could all get vaccinated, because we work with families in the community.”	8
	“With my employer…we’re working on a partnership to get vaccinated…and I got my appointment within the next week.…It had to do with the partnership, the partnership that my job had a partnership with. I think that was basically it, just knowing that the partnership that we had, it’s been an organization that’s been around for many, many years, and they do a lot of work in our community. I think that’s what was more comforting to say, ‘Okay, I think this is fine,’ versus going to some drive-up parking lot saying, ‘Come get your COVID[-19] vaccine.’”	4
	“Having the vaccine sites at some of our local churches I think would make us feel more comfortable.”	1
Support in Spanish	“He doesn’t speak English.…So, a lot of the information that he may have heard about the vaccine and COVID[-19] probably came off of Univision and Telemundo.”	4
	“There are always people who speak Spanish…do not be afraid to go to put it…the forms are given in Spanish too.”	7

### Pervasive Mistreatment of Black and Latinx Communities and Associated Distrust

Participants shared that the lingering legacy from historical mistreatment, in addition to present-day mistreatment, contributed to distrust and impacted acceptance of the COVID-19 vaccine. Disparate death rates from COVID-19 and other medical conditions, as well as ongoing experiences of their voice being “thrown away” and ignored, impacted COVID-19 vaccine acceptance.

#### Subtheme: The Lasting Legacy From Historical Mistreatment

The lasting legacy from historical mistreatment and the concern for potential reoccurrence were cited as factors influencing vaccination. A participant shared that events, such as the Tuskegee syphilis study, fostered concern for recurrent mistreatment related to COVID-19 vaccines. They remarked, “The Tuskegee experiment information in your head…that actually pulled a certain group of Black Americans and did this to them…are they setting certain vials away…to give when a Black person sits down?”

#### Subtheme: Disparate Death

Participants shared experiences of unequal death from the medical system that contributed to fears of interacting with the health care system and getting vaccinated. One participant shared, “People die from childbirth, regular colds…they’re just ignored…so, I feel that’s a huge part of moving with getting this vaccine.” Another participant expressed that vaccine concerns were related to the disparate death rates from COVID-19 in African American communities. “Knowing a lot of disparities that are with African Americans in health and seeing just the different reactions that people have with COVID[-19]…I feel like there’s just as much risk in allowing yourself to kind of get COVID[-19] as much as getting a vaccine.”

#### Subtheme: Experiences of Your Voice Being “Thrown Away” and Ignored

Participants frequently cited pervasive and ongoing mistreatment of Black and Latinx communities by the health care system as factors influencing vaccine acceptance. Mistreatments included perceptions of having their opinion “thrown away” and having their voice go unheard by the health care system. These experiences were then related to COVID-19 vaccinations. One participant explained, “I had to fight my way to get certain things done for my health. They don’t take you seriously sometimes…that kind of contributes to the fear of wanting to get vaccines.”

### Informing Trust Via Trusted Messengers and Messages, Choice, Social Support, and Diversity

Receiving information from trusted messengers, consistent messaging, fact-based information, and transparent and continued messaging informed feelings of trust in the COVID-19 vaccine. Having choices in the vaccination process, social support, and seeing diversity at the vaccination site influenced confidence and feelings of reassurance related to getting vaccinated.

#### Subtheme: Information From Trusted Messengers

Participants wanted information from trusted messengers who were reliable sources to them. “People who know the community, people who know me, I think that’s the deciding factor that would make me actually want to get it,” said a participant.

#### Subtheme: Consistent Messaging

Participants repeatedly expressed that consistent messaging was needed regarding the COVID-19 vaccine, as inconsistencies in messaging fostered confusion and lack of trust. A participant said, “It’s all confusing because none of them have the same message…makes it harder to believe anything.” Another participant remarked that “conflicting messages can be very confusing…it also adds to the mistrust.”

#### Subtheme: Fact-Based Information

Participants shared that fact-based information helped combat misinformation. One participant said, “We need to develop a platform where the positive part of the vaccine can be spreading because there’s so much negative in social media.”

#### Subtheme: Transparent and Continued Communication With the Community

Participants shared an appreciation for transparent and continued communication with the community as a way to facilitate engagement with health care practitioners and receive accurate information. “I feel like there’s just that level of transparency that’s needed between health care professionals and providers to communities like this,” said a participant. It was repeatedly expressed that communication needed to be continued with the community. A participant voiced, “The more we can have this discussion and just be honest with one another, I think the better it’s going to be.”

#### Subtheme: Increasing Confidence Through Choice

Participants shared that having a sense of personal choice facilitated confidence in the vaccination process. “There was a list of the different vaccines, and I checked the one that I wanted…I had a choice,” highlighting that there was a choice in vaccine manufacturer that was transparently listed. Another participant shared their experience about having choices in the vaccination process. They noted, “It just made me feel relieved that I had a choice of which lane I could go to.”

#### Subtheme: “Go With You”: The Power of Social Support

Support during the vaccination process, including receiving guidance for signing up and having family or community support during the vaccination, was frequently cited as a facilitator. One participant commented, “If they see a neighbor go with them…I see somebody else is in the same boat as I am, then I don’t feel so scared.” Another vaccinated participant shared with an unvaccinated participant, “I want to go with you…where it would give her some strength and some confidence.”

#### Subtheme: Reassurance in Seeing Diversity

Participants were reassured by seeing a diverse group of individuals at the vaccination site. One participant said, “When I got in there, seeing the diversity, it made me feel better.” Another participant appreciated seeing a diverse range of professionals in line for the vaccine and said, “When I went inside and seen all the policemen, doctors all in line…and I’m looking like, okay…must be for real.”

### Addressing Structural Barriers to Vaccination Access

Multiple structural barriers were identified by participants as impacting their vaccination access, including concerns about vaccine supply in Black and Brown communities being impacted by White communities, “going in circles” in the sign-up process causing fatigue, and insurance concerns. Vaccinations at schools and workplaces, with community partners and with support in Spanish, were shared as avenues to address barriers to vaccine access.

#### Subtheme: “Cutting the Line” and Vaccine Supply Access

Participants shared experiences of feeling that vaccine availability was limited in Black and Brown communities and that supply was being commandeered by White communities. One participant commented that “You hear about the White people that are going to the Black and Brown communities and cutting the line.”

#### Subtheme: “Going in Circles”: Sign-up Fatigue

Participants described inaccessible and fatiguing sign-up processes that obstructed access to vaccine appointments. One participant said, “The numbers that they were giving were not working numbers…they kept going around in circles.” Another participant stated: “You follow all these steps, and when you get to the end, it says, ‘Pick a time,’ and it goes, ‘no time available.’”

#### Subtheme: No Insurance? No Worry!

Participants shared it was important to know that insurance was not required for vaccinations. One participant proceeded to educate the focus group about this fact. “If you do have insurance, great, but if you don’t, it’s not a worry.” Another participant noted, “People are afraid to go get vaccinated, because many think that they are going to be charged to their health insurance.”

#### Subtheme: “It’s Right Here”: Schools, Workplaces, and Community Partnerships as Facilitators

Participants shared that having vaccines offered through schools and workplaces and with trusted community partners facilitated vaccination. One participant noted, “It was super easy for me because the clinic was at my school…it’s right here, no excuse.” Another participant expressed that their workplaces partnered with a trusted community organization and this served as a facilitator in getting vaccinated. “With my employer…we’re working on a partnership to get vaccinated…and I got my appointment within the next week.”

#### Subtheme: Support in Spanish

Support in Spanish is needed in every step of the process, including educational information and at the vaccination sites themselves. One participant commented that their family member did not speak English and “A lot of the information that he may have heard about the vaccine and COVID-[19] probably came off of Univision.” Another participant shared with other participants that “There are always people who speak Spanish…do not be afraid to go.”

## Discussion

This qualitative study on COVID-19 vaccine acceptance and access among Black and Latinx participants identified 3 themes with critical implications for equitable uptake of the COVID-19 vaccine. Themes included pervasive mistreatment of Black and Latinx communities and associated distrust; informing trust via trusted messengers and messages, choice, social support, and diversity; and addressing structural barriers to vaccination access. Our findings call for creating more trustworthy health care systems that are able to approach vaccine acceptance and access through the lens of equity, informed by the communities they serve. Listening and responding to the community’s needs to improve COVID-19 vaccine acceptance and access and to inform policy recommendations must remain at the forefront (Table 3). Our participants’ experiences underscore tenets of the WHO Measuring Behavioral and Social Drivers of Vaccination Increasing Vaccination model, highlighting the importance of thoughts and feelings related to trust, addressing structural barriers impeding access, and social processes, including social support, all of which may influence motivation to vaccinate.

**Table 3.** Policy Recommendations and Opportunities for Improvement to Inform COVID-19 Vaccine Acceptance and Access in Black and Latinx Communities

Themes	Opportunity for improvement
Pervasive mistreatment of Black and Latinx communities and associated distrust	Identify, address, and dismantle disparities and systems rooted in structural racism Timely and transparent release of demographic data related to COVID-19 vaccination efforts, COVID-19 deaths, and other health care–associated mortality and morbidity Measure and address critical areas of disparities in health and health care Create strategic short- and long-term plans for resource allocation informed by partnerships with community-based organizations, public health leaders, and health care systems Build community partnerships and foster bidirectional communication Create ongoing opportunities between communities and health care systems that facilitate listening and responding to the community’s needs View communities and community partners as equal stakeholders Partner with trusted organizations with ties to historically oppressed communities to create sustainable ways to increase trustworthiness now and beyond the pandemic Integrate community members and community leaders on medical boards and community advisory boards to ensure their voices inform change Invest in and foster workforce diversity, equity, and inclusion Recruit and support a diverse staff of all health care professionals that reflects the community being served Support early education pipeline initiatives to create future diverse generations of health care professionals and leaders Cultivate an understanding of past mistreatments of specific racial and ethnic communities and emphasize community informed solutions to establish equity
Informing trust via trusted messengers and messages, choice, social support, and diversity	Partner with and amplify trusted messengers, provide fact-based, consistent, and transparent information, and ensure all messaging is informed by the communities it serves Pair vaccinated community members with health care professionals to provide testimonials on the vaccination experience, incorporating culturally concordant public health messaging (utilizing guides such as Train the Trainer) Use well-known and reputable sources for information Ensure consistency in communication Update the community by sharing transparent and timely COVID-19 vaccination information Provide ongoing spaces for community dialogues that are also accessible for those with limited technological access Incorporate multiple communication modes, such as videos and storytelling, and ensure all literacy levels are represented Offer and encourage choice through every step of the vaccination process Including choice in vaccination date, time, appointment location, vaccine manufacturer, and choice of seat or vaccination lane at clinics Encourage and facilitate the use of social support throughout Encourage the use of friends, family, and community support for those who may need assistance with signing up for the vaccine or desire support on the day of vaccination Allow for social support to be present at the time of vaccination Ensure that diversity, equity, inclusion are highlighted in every step of the vaccine process Vaccination material, websites, and education must reflect the diversity of the population (in race and ethnicity, sociodemographic factors, and professions). This includes equitable and inclusive treatment of all people in the sign-up process, vaccination process, and postvaccination periods
Addressing structural barriers to vaccination access	Prioritize vaccine access to communities hardest hit by the pandemic Dynamically evaluate COVID-19 infection rates and hot spots geographically and promote vaccination in areas of larger need Continually assess vaccine distribution patterns and allocation of vaccines and prioritize distribution to hardest hit locations, including lowest income, rural, urban, and areas with highest social risk indices Consider partnering with community-based organizations, local health departments, social services agencies, and federally qualified health centers Use place-based interventions that leverage the effectiveness of community partnerships and trust (eg, convenient walk-in and pop-up sites, such as grocery stores, parks, and other highly trafficked spaces) Offer vaccination clinics after business hours and on weekends to promote vaccination of essential workers In communities with multiple vaccine sites, collaboration should occur to centralize access to ensure the community’s vaccine needs are met, not excluding communities or duplicating efforts Eliminate sign-up processes when possible Provide direct vaccine access to avoid unnecessary sign-up processes and burdensome health system navigation Use mobile community health care vans and systems to provide direct access to vaccines Have walk-in appointments and same-day scheduling in key communities with the highest social risk indices If sign-up systems are needed, ensure equity Use a myriad of modalities, including in-person sign-up (with peer navigators or community health workers to assist), functioning phone systems, and user-friendly functional online platforms with up-to-date information Create a centralized vaccination sign-up platform Ensure short wait times to eliminate barriers, such as limited phone minutes or internet access Place clear signage on all material and at vaccination sites that emphasizes no insurance is needed and that vaccines are free Signage should be inclusive, accessible to all levels of health literacy, include pictures, and use easy to understand language Partner with schools, workplaces, and other community partners to increase convenience of vaccinations Arrange for a clinician to come to places of school and work and administer vaccines to those who desire vaccination Work with trusted community partners to facilitate vaccination efforts Consider offering financial incentives for vaccination Offer paid time off after the first and second dose of vaccines as well as coverage for work-related duties Provide bilingual support at every step of the vaccination process, including written, audio, and signage at vaccine sites

A key finding of this qualitative study was pervasive mistreatment of Black and Latinx communities, rooted in structural racism, that influenced acceptance of COVID-19 vaccines. Tragedies ranging from the US Public Health Services Syphilis Study at Tuskegee to recently reported disparities in maternal mortality and COVID-19–related mortality fueled untrustworthiness in the COVID-19 vaccine and health care systems. Our participants’ perspectives are supported by systematic reviews providing data exemplifying that systemic racism and health care bias are associated with negative health outcomes and lower quality of care.^18,19^ Discrimination and inequitable treatment of Black and Latinx communities by health care systems further drive untrustworthiness related to COVID-19 vaccinations.^6,20^ Health care systems and public health organizations must understand and address the forces of structural racism that perpetuate disparities in COVID-19 vaccinations and health inequities.^9,21,22^

Informing trust via trusted messengers and messages, choice, social support, and diversity is critical to fostering trustworthiness and vaccine acceptance.^6,23,24,25,26,27^ Trusted messengers include individuals regarded as credible by their community, such as health care professionals, faith leaders, and community stakeholders. Public health agencies and health care systems stand at an opportune time to form authentic, long-standing, and mutually beneficial alliances with trusted messengers and key community stakeholders to help broker trust based on the community’s earned reliability and respect. Partnerships with trusted messengers, communities, and their stakeholders can serve as conduits to deliver accurate, consistent, and transparent messaging from trusted sources, which has been cited as an effective tool for increasing vaccine acceptance and other beneficial health behaviors.^6,23,24,28,29,30,31^ The community holds the expertise of what their needs are and what interventions are likely to succeed. Health care systems often hold valuable resources that benefit the community if used effectively.^31^ Combining such strengths are critical and have been successful against tobacco use (eg, the national “Truth” campaign) and with the Vaccinate with Confidence program, a framework that promotes protecting communities, empowering families, and stopping myths.^28,29,30,32,33^

Offering choice in the vaccine process was perceived as facilitating a sense of control and influencing willingness to receive a COVID-19 vaccine.^34^ Choice should be offered in as many aspects of the vaccination process as possible to facilitate trust and transparency. This includes choice in vaccine manufacturer, a mix of community and health care–based vaccination sites, and allowing individuals to choose the lane and chair they select to be vaccinated in at the time of vaccination.

The influence of social support is a known tool in increasing behaviors to vaccinate.^35,36,37,38^ The support of family, friends, and one’s local community are critical to vaccine acceptance, as decisions related to health behaviors are often drawn from patterns within small communities.^39^ Additionally, vaccination sites should reflect the diversity of the population, including race, ethnicity, and profession, which is imperative to fostering trust. Equally important is ensuring that equitable and inclusive care is delivered to all.

Structural barriers underscore the narrative of inequitable access to COVID-19 vaccinations and point to the larger health care system changes needed to ensure equity. Vaccine distribution patterns must be analyzed and reassessed to ensure distribution is adequate and consistent to communities with the highest social risk indices and those hardest hit by the pandemic.^40,41^ To further remove barriers, mobile clinics, same-day clinics, and walk-in vaccination sites in easily accessible and trusted locations can be used.^42^ If sign-up systems must be used, ensure multimodal access.^43,44,45,46^ Highlighting the no-cost nature of the vaccine and removing documentation requirements are essential to eliminating barriers.^46^ We stand at a time when overcoming vaccination barriers will not only determine whether disparities in outcomes for Black and Latinx communities are worsened but also determine whether vaccination efforts are able to sufficiently mitigate the progression of the pandemic through variants in the coming year.^6^

### Limitations

This study has some limitations. Our study intentionally included participants who identified as Black or Latinx from a medium-size city. Experiences may not represent that of other communities, such as rural populations or other racial and ethnic populations. Our study provides the perspectives of both Black and Latinx communities, and nuanced perspectives of each individual community may not be captured. Findings may be susceptible to social desirability bias. We did not include segmentation by age, vaccines status, or occupation, and required the use of video conferencing software owing to pandemic social distancing precautions, which may change levels of participation compared with in-person groups.

## Conclusions

The findings of this qualitative study suggest that key community-informed levers may have the potential to impact COVID-19 vaccine acceptance and access among communities hardest hit by the pandemic. These findings may impact public health initiatives and health care systems’ ability to reach Black and Latinx communities to increase equitable uptake of the COVID-19 vaccine and ensure health for all communities.
